# Preparation and Tribological Study of Biodegradable Lubrication Films on Si Substrate

**DOI:** 10.3390/ma8041738

**Published:** 2015-04-14

**Authors:** Shih-Chen Shi, Teng-Feng Huang, Jhen-Yu Wu

**Affiliations:** Department of Mechanical Engineering, National Cheng Kung University (NCKU), No.1 University Road, Tainan 70101, Taiwan; E-Mails: N16031760@mail.ncku.edu.tw (T.-F.H.); N16031794@mail.ncku.edu.tw (J.-Y.W.)

**Keywords:** green, tribology, water-degradable, hydroxypropyl methylcellulose, HPMC, lubrication, friction coefficient, wear, ball-on-disk

## Abstract

A novel method for preparing eco-biodegradable lubricant based on hydroxypropyl methylcellulose (HPMC) via hydration process is demonstrated. The smooth and homogeneous HPMC coating has a uniform thickness (~35 μm). It has been demonstrated that the preparation parameters play a critical role in controlling the lubricating behavior of the coating; in addition, excess HPMC and water concentration suppress the tribology properties. Nevertheless, a remarkable friction-reduction and anti-wear performance has been obtained. Impressively, the preparation parameter of 5% HPMC + 30 mL water significantly improves lubricant performance and durability. A simple approach for the water-degradability evaluation of HPMC is proposed.

## 1. Introduction

Microelectromechanical system/Nanoelectromechanical system (MEMS/NEMS) is a technology combining electronic and mechanical devices by using a mature semiconductor processing technique in the scale of micron/nanometer. NEMS/MEMS products are successfully entering the market and created a thousand billion US dollars in revenue in the year of 2014. It has been treated as a power source of the next industrial revolution. However, severe wear occurring in silicon materials has been a bottleneck for wide application. Therefore, a very effective solution to improve MEMS durability is to find a novel material with potential tribology performance [1,2].

In the past two decades, environment and energy issues at a global scale have increased seriously year by year. However, very recently, there has been increased activity in the development of green technology, with quite a few reports on bio/eco technology. Biofriendly synthesis techniques have been reported [3,4,5,6]. Biofriendly materials, such as silica gel, functionalized gold nanoprobe and xylene substitute, have been prepared [7,8,9]. Biodegradable materials and application also make their appearance [10,11]. Applications of biodegradable lubricants have been reported [12,13,14,15]. Moreover, biodegradable devices have also been announced [16,17,18,19].

Manufacturing enterprises ask for lubricants without phosphorus or heavy metals as well as non-toxic substances for the reasons of environmental and toxicological considerations. For this reason, tribology techniques expand into the direction of saving energy, reducing materials usage and waste, decreasing noise and shock, increasing recycling, and developing environmentally friendly lubrication [20,21,22,23]. This idea creates an opportunity to produce environmentally friendly, environmentally acceptable, biofriendly and even biodegradable lubricants from natural substances. The benefits of green tribology products include depressed pollution, minimum hazardous risks, and easier reuse due to their biofriendly properties. Green tribology lubricants should be non-toxic and harmless to the human body through contact and inhalation. Due to environmental and toxicological considerations, the use of natural materials, such as vegetable, soy bean and even coffee bean, as a lubricating oil is prompted [24,25]. It is easy to improve their tribological performance by natural additives, such as sugar cane, to fulfill the green tribology requirements [26,27,28,29,30]. Stable additive materials. including layered transition metal dichalcogenide [31,32,33], graphene [34,35], diamond [36,37,38], metal nanoparticle [39] and oxides/nitride [40,41], are potential additives to improve tribology performance as well.

Numerous important applications of cellulose derivatives, including pharmaceutical, food, adhesive and textile fields, have been widely studied for many years. Cellulose automatically qualify as an ideal polymer in the formation of film coating [42,43]. High purification cellulose is marketed as the basis of additives in food, drug coating and cosmetics. Cellulose polymer is often used to control on drug release rate, especially in capsule and tablet, from controlled release formulations [44,45]. Cellulose is an organic compound, which is tasteless, odorless and non-toxic. There are many different structures of cellulose that can be applied in different fields, including methylcellulose, hydroxypropyl cellulose (HPC), and hydroxypropyl methylcellulose (HPMC). Among them, HPMC is the most widely used due to its good film formation, flexibility and ease of handling. Currently, HPMC is primarily used as a protection coating for drugs and supplements, and serves as the outer hard coating to control the ingredient release and protect the product against light and moisture. However, HPMC has certain limitations in protection coating applications because of its poor wear resistance [46]. Additives have been studied to improve the surface properties of HPMC by incorporating nano-fibers, fatty acids and essential oils and even sorbitol and chitosan [47,48,49,50].

The surface properties of HPMC play an important role when the coatings are used in domains such as protection layers. Nevertheless, very few studies concern the protection and anti-wear property of HPMC film. In this study, we focus on understanding the effects of hydration on the coating characteristics of the formulation films. Moreover, we report on the application of a biofriendly HPMC coating as an anti-wear lubricant. A novel method is demonstrated for HPMC coating manipulation to enhance the tribological performance. Furthermore, a simple approach to identify the water-degradability of HPMC is proposed and discussed.

## 2. Results and Discussion

### 2.1. HPMC Coating Preparation and Characteristic Analysis

Raw material HPMC particles (Table 1), ethanol, and water were placed in a glass container and then stirred while heated. After the reaction, the system was cooled down to room temperature and the product was injected into the silicon substrate.

**Table 1:** Specifications of hydroxypropyl methylcellulose (HPMC) powder used.

Molecular weight Mn	Methyl (CH_3_) Substitution (%)	Hydroxypropyl (CH_2_CHOHCH_3_) Substitution (%)	Viscosity (2 wt% Aqueous Solutions at 20 °C)
35,600	28.8	9	5.94 mPa·s

The sample preparation parameters are shown in Table 2. In the first stage, HPMC content is varied by fixing the ethanol concentration. After picking up the optimized HPMC content, in the second stage, volume of DI-water is the variable, by fixing the HPMC content and ethanol concentration. 

**Table 2:** Samples preparation parameters and film thickness.

Film composition	HPMC (g)	Ethanol (mL)	DI Water (mL)	Film Thickness (μm)
2% HPMC	2	100	0	35.1 ± 1.2
3% HPMC	3	100	0	35.2 ± 1.5
5% HPMC	5	100	0	35.6 ± 1.1
7% HPMC	7	100	0	35.8 ± 0.9
5% HPMC + 30 mL DI water	5	100	30	34.9 ± 1.6
5% HPMC + 50 mL DI water	5	100	50	35.0 ± 1.5
5% HPMC + 60 mL DI water	5	100	60	33.9 ± 1.7

**Figure 1:**
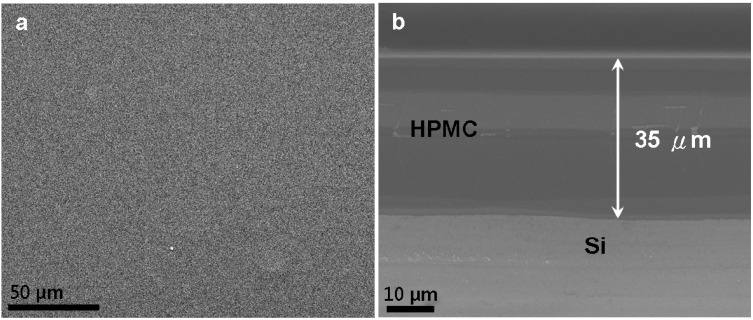
SEM images of the HPMC coating: (**a**) top-view image and (**b**) cross-sectional image prepared on silicon substrate.

Figure 1a show s a typical scanning electron microscopy (SEM) image of the morphology of the as-prepared HPMC on silicon substrate. Five percent HPMC exhibits a very smooth, flat and uniform surface morphology. A typical cross-sectional SEM image of HPMC coating is shown in Figure 1b. This image displays a dense and homogeneously distributed film over the entire substrate. HPMC coating exhibits a mean thickness of 35 μm. The beauty of this HPMC coating for anti-wear tribology technique lies in the easy thickness and location control of the film, which can be achieved simply by adjusting using volume keeping the other preparation parameters, such as ethanol and temperature, fixed. However, the stirring temperature and time period are two important and critical parameters that should be well controlled.

**Figure 2:**
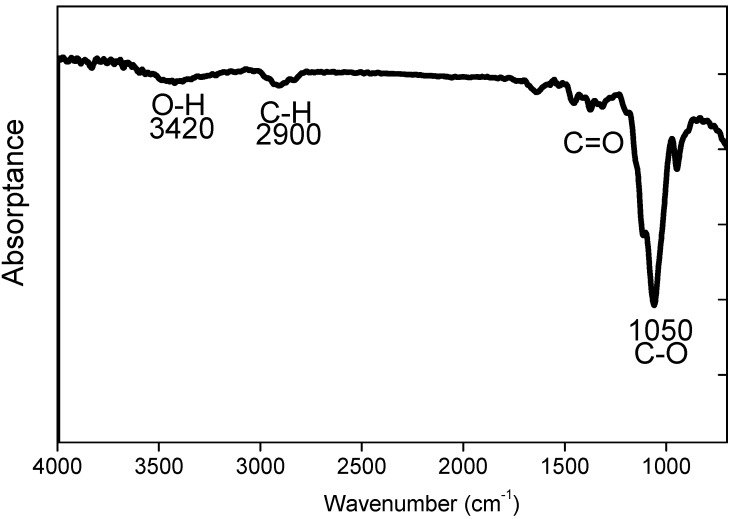
FTIR spectrum of HPMC coated on a silicon substrate. FTIR was measured from a sample prepared with 5% HPMC.

The Fourier transformed infrared (FTIR) spectra in Figure 2 gives the characteristic absorption of the HPMC coating. The main absorption band of HPMC is located at 1050 cm^−1^ (C–O stretch vibration) and smaller peaks are found between 1200 and 1500 cm^−1^. These smaller peaks overlap with the carboxylic acid groups. The bands around 1600 cm^−1^ correspond to the bending mode of water. The other major bonds are found at 2900 cm^−1^ and mainly due to absorptions of the C–H stretch bond. O–H stretch bands were found at 3420 cm^−1^. These FTIR peaks are signatures of pure HPMC as reported previously [30]. Compared to the Waara report, FTIR results exactly match with that of pure HPMC. This result demonstrates the successful preparation of HPMC coating on silicon substrate.

### 2.2. Microtribology Test

**Figure 3:**
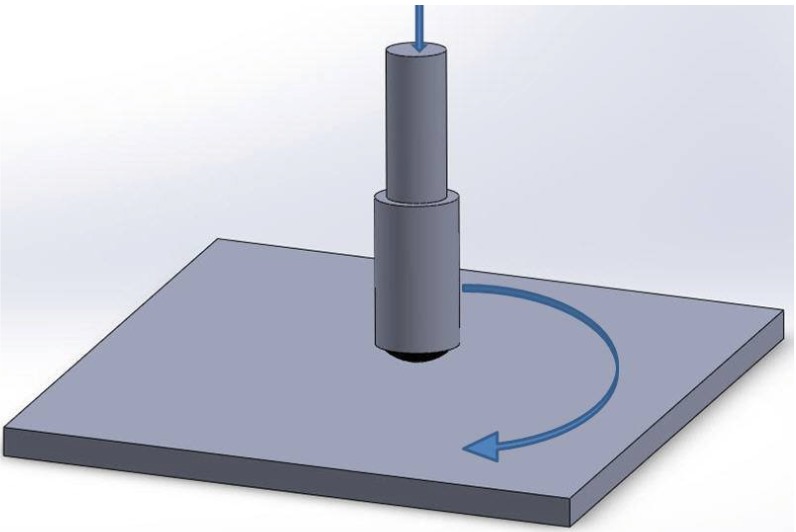
Diagram of the experimental setup that was used in the work to study the tribological properties of HPMC. Ball-on-disk tribometer: friction and wear of the various preparation parameters of HPMC lubricants were tested under low sliding velocity and load. The load (N) and velocity (V) were fixed in a predetermined setup, and the friction coefficient was recorded.

HPMC is soluble in cold water and some organic solvents. In this study, HPMC solution was prepared by dissolving HPMC powder into ethanol and water. First stage, we check the lubrication properties by using 2–7 g HPMC. Secondly stage, we want to optimize the lubrication property by adding water to the solution to enhance the tribology performance. Three parameters were set by different water concentrations, varying from 30 to 60 mL. Tribological tests were carried out for all the HPMC preparation parameters, using a ball-on-disk tribometer (Figure 3). The variation in friction coefficient *vs.* cycles can be used to determine the lubricating behavior during the tribological test.

**Figure 4:**
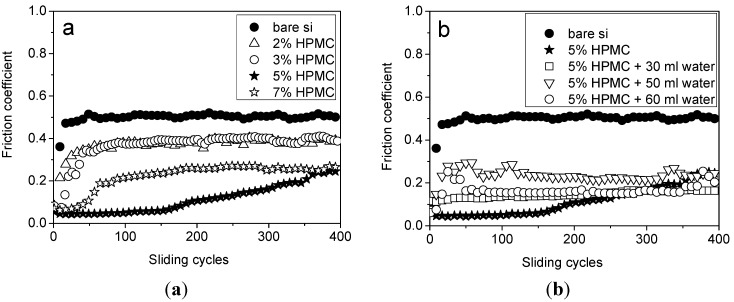
The variation of friction coefficient *vs.* cycles (**a**) with 0%, 2%, 5%, 7% HPMC, respectively; (**b**) with bare silicon, 5% HPMC, 5% HPMC + 30 mL water, 5% HPMC + 50 mL water, and 5% HPMC + 60 mL water, respectively.

Figure 4a shows the variation of the friction coefficient with cycles for five HPMC (0% (bare silicon), 2%, 3%, 5%, and 7%, respectively). The contact force and sliding velocity were fixed during each testing cycle. The friction coefficient jumps to 0.5 after 20 cycles with direct contact on silicon surface. Applying HPMC coating on silicon surface can dramatically reduce the friction coefficient. It is noticeable that lower HPMC content, such as 2% and 3%, has a 30% reduction in friction coefficient. However, the friction coefficient can be cut down by 60% with a higher HPMC content, such as 5% and 7%. Moreover, the amplitude of the friction coefficient is very low and increases gradually for the sample with 5% HPMC content. This result demonstrates that HPMC coating has a considerable beneficial effect on lubrication. However, the sample of 7% HPMC content do not work better in reducing the friction coefficient than the sample of 5% HPMC content; it is just half the magnification of friction coefficient compare to the bare silicon. It is proposed that the 7g HPMC content is too high to dissolve in the ethanol. Undissolved HPMC particle was found in HPMC-ethanol solution when HPMC content is too high. It causes deleterious effects to the lubricating performance. As a result, the trend shown in Figure 4a indicates that the friction coefficient is significantly lowered and wear is successfully suppressed by adding a suitable concentration of HPMC. It is suggested that the water content in HPMC coatings affect the lubrication behavior due to the water solubility and hydration effect. Therefore, water content is another important criterion for evaluating the tribological property. Variation of the friction coefficient by varying water concentration with cycles for five parameters—bare silicon, 5% HPMC, 5% HPMC + 30 mL water, 5% HPMC + 50 mL water, and 5% HPMC + 60 mL water—are demonstrated in Figure 4b. The trend is obviously shown when the test cycles finished 400 cycles; friction coefficient is successfully suppressed by adding water into the HPMC coating. It is surprising that the 5% HPMC + 30 mL water exhibits a much lower, stable, and consistent friction coefficient than that of the two other water-added samples. This result indicates that water concentration in HPMC coating has a positive effect on lubricating behavior when the proper concentration of water is added. However, there is no big friction coefficient difference between the 5% HPMC and 5% HPMC + 30 mL water after 400 cycles.

### 2.3. Stability and Duration Test

**Figure 5:**
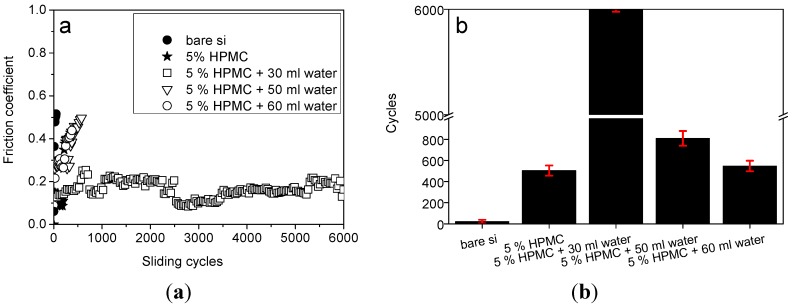
(**a**) Duration test of bare silicon, 5% HPMC, 5% HPMC + 30 mL water, 5% HPMC + 50 mL water and 5% HPMC + 60 mL water, respectively; (**b**) comparison of duration cycles of varying HPMC preparation parameters with a discontinuous vertical coordinate.

To further understanding the tribological performance of HPMC coating on long-term lubricating behavior, duration and stability tests are carried out. The evolution of the friction coefficient of 5% HPMC with different water concentration *vs.* cycles is reported. The variation in friction coefficient *vs.* long-term cycle is shown in Figure 5a. This test can be used to identity the stability of the coating during the tribological test. Figure 5a shows the variation of the friction coefficient *vs.* cycles for five preparation parameters—bare silicon, 5% HPMC, 5% HPMC + 30 mL water, 5% HPMC + 50 mL water, and 5% HPMC + 60 mL water, respectively. For bare silicon, the friction coefficient jumps very fast due to absence of lubricating coating to protect the surface and leads to a high friction coefficient. For 5% HPMC coating, thre friction coefficient increased to 0.4 gradually as the test reachers 500 cycles. With regards to 5% HPMC + 30 mL water, the friction coefficient is low from the start of the experiment and it remains stable throughout the test, even when the test completes 6000 cycles. However, higher water concentrations do not further reduce the friction coefficient more than the 30 mL water concentration sample. We suppose that the concentration of water is too high to soften the coating and lose the protection property. HPMC coating is easily worn out due to the softening behavior of adding too high a water concentration. Consequently, a high friction coefficient is observed when there is a breakdown or absence of protecting coating. The comparison of duration cycles *vs.* HPMC preparation parameters are shown in Figure 5b. Here, a friction coefficient of 0.4 was set as a cutting limit to check the long-term tribological stability of the coatings. The results shown in Figure 5a,b reveal that 5% HPMC + 30mL water is the most favorable in both reducing coefficient and suppressing wear. This indicates that a suitable water concentration in HPMC has a considerable beneficial effect in lubrication.

**Figure 6:**
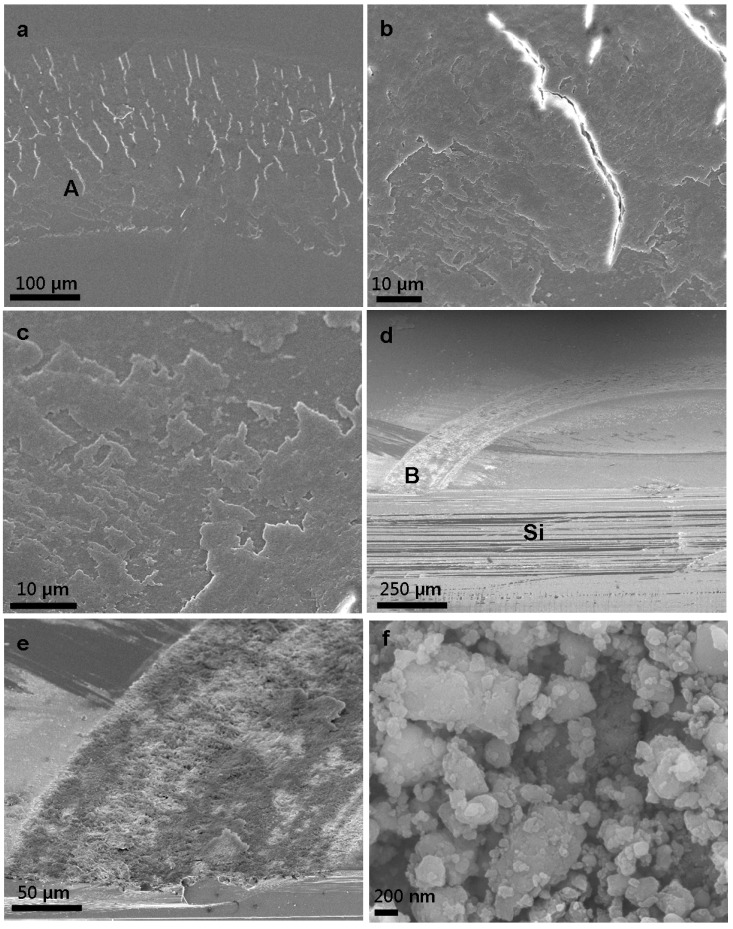
Typical SEM images after tribological duration test: (**a**) top-view image of HPMC; (**b**) enlarged part A in (**a**); (**c**) high magnification of residue HPMC coating on silicon substrate after 6000 cycles; (**d**) typical cross-sectional image of bare silicon after 20 cycles; (**e**) enlarged part B in (**d**); and (**f**) high magnification images of silicon surface after 20 cycles.

The SEM images in Figure 6a–c demonstrate the typical top-view surface morphologies of 5% HPMC + 30 mL water after 6000 cycles with various magnification. We can easily visualize the wear trace after the tribological test. White scars reveal the breakage of the protection layer after long-term wear. Figure 6c shows a clear image of the presence of a protective coating and appearance of silicone substrate; it is because the protection layer is consumed during the duration test and leads to an instable friction coefficient, especially in the latter part of the test. Typical SEM images of non-coated, worn silicon surface are shown in Figure 6d–f. The images represent that a uncoated silicon reveals serious wear track and have lots of irregular debris from the size of hundreds of nanometer to a few micrometer, even after only 20 cycles. According to these figures, severe abrasive wear is the dominant wear mechanism of the uncoated bare silicon. Compared with the post-test surfaces shown in Figure 6c,f, which are the surface morphology of 5% HPMC + 30 mL water after 6000 cycles and bare silicon after 20 cycles duration test, HPMC coating display very excellent friction coefficient reduction and anti-wear ability.

**Figure 7:**
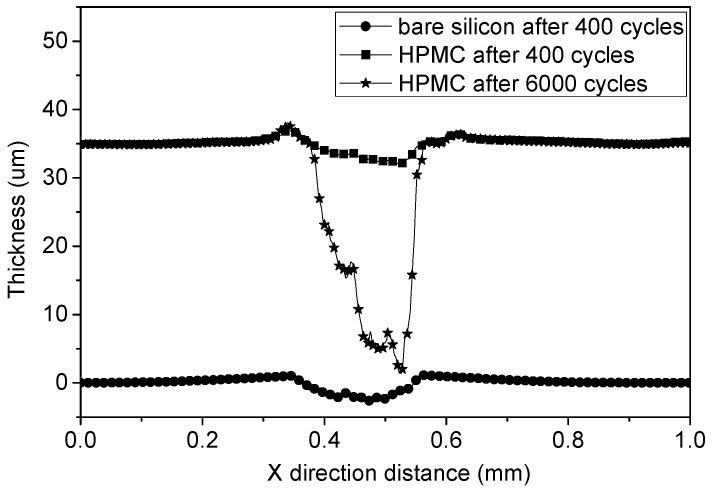
Wear performance of bare silicon after 400 cycles, 5% HPMC + 30 mL water film after 400 cycles and 5% HPMC + 30 mL water film after 6000 cycles.

Figure 7 shows the wear behavior of the bare silicon and HPMC coated silicon. For bare silicon substrate, it reveals a hole with 3.5 μm in depth. This result coincides with the evidence of Figure 6d–f. Severe abrasive wear occurred on the uncoated substrate. However, for the 5% HPMC + 30 mL water coated silicon substrate, there is no damage observed on silicon surface. This result matches well with the evidence of Figure 6c. The residual HPMC coating can be found on the silicon surface after 6000 cycles.

### 2.4. Water-Degradability Evaluation

**Figure 8:**
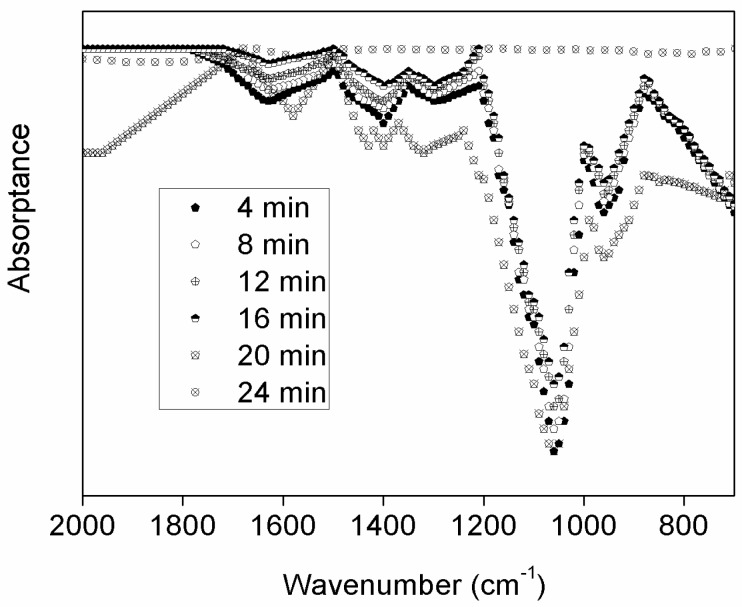
Typical FTIR spectra taken at six different stages of soaking time of HPMC in water.

In Fahs’ study, HPMC is partially soluble in water [51] and well soluble in some organic solvents [52]. This is a reversible process. Here, we propose a direct method to identify the water-degradability of HPMC by simply immersing the prepared HPMC coating into water. It is expected that the HPMC coating dissolve layer by layer with time. The FTIR spectrum of HPMC with six different soaking times in water is obtained (Figure 8). The characteristic peak at 1050 cm^−1^ shows no difference in the range of soaking time from 4 min to 20 min. When the soaking time reaches 24 min, the characteristic peak vanishes. It is expected that the HPMC coating will totally dissolve in water exposing the silicon surface beneath. As a result, there is no FTIR signal for the silicon surface. This finding is reasonable, since the penetration depth of FTIR is only around a few micrometers. To further understanding the mechanism, microtribology tests were carried out using the samples that had a soaking time longer than 24 min. It reveals a similar behavior to bare silicon (Figure 4a). Based on the above result, water-degradability of HPMC is confirmed.

## 3. Experimental Section

### 3.1. HPMC Material and Film Preparation

Hydroxypropyl methylcellulose (HPMC) powder with a hydroxypropoxy content of 9% and viscosity of 6 mPa·s was obtained from Shin-Etsu Chemical Co., Ltd., Tokyo, Japan. Ethanol (95%) was used to improve hydration of HPMC, and help to decrease the bubbles in the coating-forming process and eventually in drying the coating formation. HPMC coating was prepared by dissolving 5 g of HPMC in a solution of ethanol (100 mL) and distilled water. The solution was stirred for 30 min at 55 °C by using a heating and stirring hot plate. After a homogenous solution was achieved by the stirring and mixing process, films were made by a pipette injection on the silicon substrate and left to dry at room condition: 25 ± 2 °C and 60% ± 5% related humidity (RH) for 24 h. Micropipette (VITLAB 11M99651, Grossostheim, Germany, VITLAB) was used for precise volume-control during the injection process. The thickness of the films was measured using cross-sectional SEM images. The thickness was measured in 10 randomly selected samples, as shown in Figure 1b.

### 3.2. Surface Observation by Scanning Electron Microscopy (SEM) and Surface Recorder

The surface morphology was characterized using high-resolution thermal field emission scanning electron microscopy (JSM7001F, Tokyo, Japan, JEOL Ltd., acceleration voltage under 10 kV, probe current of 100 nA, working distance of 10 mm), including top view and cross-sectional image. Wear phenomenon was recorded using microfigure measuring instrument (Surfcorder ET3000, Tokyo, Japan, Kosaka Laboratory Ltd.).

### 3.3. Fourier Transform Infrared Spectrometer Analysis

Fourier transform infrared spectrometer (Thermo Nicolet NEXUS 470 FTIR, Golden Valley, MN, USA, GMI) was used to study the structural properties of HPMC coatings with a software-assisted interface (OMNIC). The spectra were obtained by reflection mode in scanning speed of 50 scans/s with the scanning range of 500–4000 cm^−1^.

### 3.4. Microtribology Test

This study designed to investigate the effect of the HPMC structure and preparation parameters on friction and wear, was done and by using a ball-on-disk tribometer (Figure 1). Test setup was a ball fixed on a stationary holder, and the bottom disk rotated with a specified speed. Microtribology test were performed in ambient air (50% RH) and ambient temperature (25 °C) with a sliding speed of 0.01 m/s and rotate radius of 2 mm. All friction and wear tests were conducted at a rotating speed of 95 rpm and loads of 2 N. The steel balls (radius 3/32 inch) are made of DIN 17350 (100Cr6) with a hardness of 61–64 HRC. The tested bio-friendly HPMC coating was prepared on the silicon substrate and mounted onto the tribometer bottom disk. Resistance to the motion of the disk (*i.e.*, friction force) was measured by a load cell connected to the rotating disk. The friction coefficient was recorded for further tribological property analysis. The friction experiments were repeated to ensure reproducibility of the results. 

### 3.5. Water-Degradability Evaluation

The water-degradability of HPMC coating was measured by monitoring the characteristic peak signal of HPMC in FITR. The HPMC coated (5% HPMC + 30 mL water) silicon substrates were prepared and immersed into DI-water. The test was carried out at environment temperature of 25 °C for 28 min.

## 4. Conclusions

The tribological performance of HPMC coating on silicon has been demonstrated. Lubricating behavior is highly related to the weight percent of HPMC and water concentration. It is further shown that by adding suitable HPMC and water concentration, the friction coefficient can be significantly lowered at greater stability. The HPMC coating is a promising lubricant material with great friction-reduction and anti-wear characteristics, especially in the green tribology and MEMS applications. FTIR technology was performed to analyze the structure and water-degradability of HPMC. In this study, we report on the first application of biofriendly HPMC coating lubricating behavior. A novel approach for HPMC coating manipulation to enhance the tribological performance is demonstrated.
